# Impact of biologic and targeted synthetic therapies on bone health in immune-mediated inflammatory diseases: a narrative review

**DOI:** 10.3389/fmed.2026.1854661

**Published:** 2026-08-26

**Authors:** Isabel de la Cámara-Fernández, Tatiana Cobo-Ibáñez, Olga María Reillo-Sánchez, Gabriela Nataly Cueva-Nájera, Ana Valeria Acosta-Alfaro, Santiago Muñoz-Fernández

**Affiliations:** 1 Department of Rheumatology, Infanta Sofia University Hospital, Madrid, Spain; 2 Department of Medicine, Faculty of Medicine, Health, and Sports, Universidad Europea de Madrid SLU, Madrid, Spain; 3 FIIB HUIS-HUHEN, Madrid, Spain; 4 Infanta Sofia University Hospital, Madrid, Spain

**Keywords:** biologic therapies, bone density, fractures, immune-mediated inflammatory diseases, osteoporosis, targeted synthetic therapies

## Abstract

**Background:**

Osteoporosis is a systemic skeletal disorder characterized by reduced bone mass, impaired microarchitecture, and increased fracture risk. Patients with immune-mediated inflammatory diseases (IMIDs) have a higher prevalence of osteoporosis owing to chronic inflammation, glucocorticoid use, and disease-specific factors. Pro-inflammatory cytokines such as TNF-*α*, IL-1, IL-6, and IL-17 contribute to bone loss, suggesting that therapies targeting these pathways may influence skeletal health.

**Objective:**

This narrative review aims to evaluate the effects of biological disease-modifying anti-rheumatic drugs (bDMARDs) and targeted synthetic DMARDs (tsDMARDs) on bone metabolism and fracture risk in adults with IMIDs.

**Methods:**

A comprehensive literature search was conducted in Ovid Medline, Embase, and the Cochrane Central Register of Controlled Trials from 2015 to August 2025, using MeSH and free-text terms. Reference lists of included studies were manually screened. Search strategies were validated by an expert librarian.

**Results:**

bDMARDs and tsDMARDs, including TNF-*α* inhibitors, IL-6 inhibitors, abatacept, rituximab, IL-17 inhibitors, and JAK inhibitors, generally preserve or modestly increase bone mineral density (BMD) and favorably modulate bone turnover markers (BTMs), although these effects do not always correlate with changes in BMD. However, in some cases, a decrease in BMD has been reported; this is usually associated with persistent inflammatory disease activity. Despite these effects, evidence for fracture risk reduction is limited and inconsistent, with outcomes influenced by disease activity, structural damage, and classic risk factors.

**Conclusion:**

bDMARDs and tsDMARDs appear to preserve or modestly improve bone mass and positively influence bone turnover in IMIDs. However, increased BMD does not consistently translate into lower fracture risk, and current evidence is insufficient to draw definitive conclusions regarding the effect of bDMARDs and tsDMARDs on fracture risk.

## 1 Introduction

Osteoporosis is a systemic disease characterized by decreased bone mass, altered bone microstructure and microarchitecture (bone quality), and, consequently, an increased risk of fragility fractures. Its etiology is multifactorial, involving endocrine, metabolic, mechanical, and immunological factors, and it can be secondary to treatment and other diseases.

Osteoporosis is more prevalent in patients with immune-mediated inflammatory diseases (IMIDs) than in healthy controls (1, 2). A recent umbrella review found the prevalence of osteoporosis in chronic autoimmune diseases to be as follows: rheumatoid arthritis (RA), 27.6% (95% CI: 23.9–31.3); systemic lupus erythematosus (SLE), 16% (95% CI: 12.0–19.0); systemic sclerosis, 27% (95% CI: 24.0–31.0); multiple sclerosis, 17% (95% CI: 14.0–20.0); and coeliac disease, 14.2% (95% CI: 9.0–20.5). In addition, the prevalence of osteoporosis was estimated to be 22.5% (95% CI: 11.7–34.4) in axial spondyloarthritis (axSpA) (3). Another meta-analysis recorded a prevalence of 14.64% (95% CI: 11.21–18.89) among patients with systemic vasculitis (1) and 12.2% (95% CI: 9.1–15.3) among patients with inflammatory bowel disease (IBD) (2). An increase in the prevalence of low bone mass has also been reported in inflammatory myopathies, although the results are based on small samples (4, 5). Several studies have reported an increased prevalence of osteoporosis in patients with primary Sjögren’s syndrome. Smaller studies suggest that up to one-third of patients are affected, while a larger cohort study of 437 patients reported a prevalence of 18.5% (6, 7).

Several studies have shown that the presence of osteoporosis is conditioned by the specific IMID (e.g., nephritis in SLE), by disease-related factors (e.g., use of glucocorticoids), or by other factors (e.g., menopause and immobility) (8–14).

IMIDs are characterized by persistent activation of the immune system, leading to chronic inflammation. This inflammatory state plays a central role in the development of secondary osteoporosis through complex osteo-immunological mechanisms that alter the balance between bone formation and resorption. Some of the cytokines involved in these mechanisms, such as TNF-*α*, interleukin 1 (IL-1), interleukin 6 (IL-6), and interleukin 17 (IL-17), also play a central role in the pathophysiology of various IMIDs (15). Therefore, blocking these cytokines with biological disease-modifying anti-rheumatic drugs (bDMARDs) and targeted synthetic DMARDs (tsDMARDs) may constitute a fundamental therapeutic strategy, not only by attenuating the inflammatory activity of the primary disease, but also by preserving or even improving bone mineral density (BMD), which is often compromised in IMIDs. This protective effect is clinically relevant, as stabilizing or increasing bone mass leads to a significant reduction in the risk of fractures (16), thereby decreasing the morbidity and mortality associated with bone complications, without forgetting that we must adopt a holistic approach, also acting on the other factors that influence bone metabolism and fracture risk, in order to achieve the best possible bone health in patients with IMIDs.

## 2 Objectives

This review aims to understand the risk of bone fragility and the role of osteo-immunology in IMIDs. It also aims to examine scientific evidence for the effect of bDMARDs and tsDMARDs on bone metabolism in adults with IMIDs, assessed based on bone turnover markers (BTMs) and BMD, and to evaluate the influence of these drugs on fracture risk.

## 3 Bone fragility in immune-mediated inflammatory diseases

The risk of low bone mass, osteoporosis, and/or fragility fractures may be increased in patients with IMIDs owing to traditional risk factors, although disease-specific risk factors also play a role. Several studies have identified disease-specific risk factors for RA (14, 17–20), axSpA (14, 21–23), psoriasis and psoriatic arthritis (PsA) (24, 25), SLE (26–29), primary Sjögren’s syndrome (7), systemic sclerosis (30, 31), systemic vasculitis (1), and IBD (2, 32). These risk factors are shown in Table 1. Other IMIDs have also been associated with reduced bone mass and increased fracture risk compared with reference populations. However, specific risk factors for osteoporosis in these conditions have not yet been clearly identified.

**Table 1:** Disease-specific risk factors associated with low bone mass and fractures in IMIDs.

Disease	Bone involvement/fracture risk	Risk factors for low bone mass	Long-term fracture risk factors	Additional considerations
Rheumatoid arthritis	Increased risk of low BMD, osteoporosis, and fractures	- ACPA positivity (14, 20)	- Low BMI (≤20 kg/m^2^) (17, 18)	ACPA positivity is associated with lower BMD. Rheumatoid factor and ACPA positivity increase the 10-year fracture risk compared with seronegative patients (14, 19, 20).
			- Failure to achieve remission or low disease activity during the first 2 years (18)	
			- High disability (HAQ ≥ 1) in the first year (18)	
			- Early progression of bone erosions (within the first 2 years) (18)	
			- Corticosteroid therapy (dose and cumulative exposure) during the first 2 years (17, 18)	
			- Rheumatoid factor and ACPA positivity (18, 19)	
Axial spondyloarthritis	Loss of trabecular bone; increased risk of vertebral fracture	- Bone marrow edema on MRI (even in early and nr-axSpA) (14, 22, 23)	- Men (21, 22)	Greater risk of vertebral fractures than in the general population.
			- Low BMD (21)	
			- Longer disease duration (21)	
			- Radiographic damage (BASRI) (21)	
Psoriasis/Psoriatic arthritis	Increased risk of fracture without a strong association with decreased BMD	- Chronic systemic inflammation; possible alterations in bone microstructure (25)	No significant relationship was observed between psoriasis/PsA and the risk of osteoporosis (24, 25)	Fractures may occur despite normal BMD; DXA may underestimate bone fragility owing to impaired bone quality
Systemic lupus erythematosus	Increased risk of bone loss (especially lumbar spine) and fractures	- Advanced age (26, 27, 29)	Risk factors for vertebral fractures	Increased risk of femoral neck fractures and vertebral fractures has been reported, although up to 35.8% of patients with vertebral fractures present with normal BMD (27)
		- Longer disease duration (28, 29)	- Mean age around 50 years (27)	
		- Cumulative glucocorticoid dose and duration (28, 29)	- Low total hip BMD (27)	
		- Higher SLICC damage index (29)	Risk factor for femoral neck fracture (26)	
		- Menopause (28, 29)	- Age ≥40 years	
			- Use of intravenous cyclophosphamide	
			- Higher dose of corticosteroid	
			- Stroke	
			Risk factor for trochanteric hip fracture (26)	
			- Age ≥50 years	
Primary Sjögren’s syndrome	Increased risk of osteoporosis and fractures	- Postmenopausal status	- Age >64 years	All risk factors obtained from reference 7
		- Age >64 years	- Disease duration	
		- Longer disease duration	- Disease activity measured by ESSDAI	
		- Prior corticosteroid treatment		
Systemic sclerosis	Increased risk of osteoporosis	- Postmenopausal status (30)		
		- Age >50 years (30)		
		- Aging (30, 31)		
		- Longer disease duration (30)		
		- Anti-topoisomerase I antibodies (31)		
		- Glucocorticoid treatment (31)		
Systemic vasculitis	Increased risk of osteoporosis	- Cumulative glucocorticoid exposure (dose-dependent relationship) (1)		
Inflammatory bowel disease	High prevalence of osteoporosis; increased fracture risk	- Corticosteroid therapy (2)		Similar risk in Crohn’s disease and ulcerative colitis. Increased fracture risk up to 32%, but specific risk factors not identified
		- Malnutrition (2)		
		- Disease activity (2)		
		- Genetic factors (2)		
Other IMIDs (e.g., multiple sclerosis, inflammatory myopathies, autoimmune thyroid disease, celiac disease)	Reduced bone mass and increased fracture risk compared with reference populations			Specific risk factors for osteoporosis in these conditions have not been clearly identified.

IMIDs, immune-mediated inflammatory diseases; BMD, bone mineral density; ACPA, anti-cyclic citrullinated peptide antibody; BMI, body mass index; HAQ, Health Assessment Questionnaire; MRI, magnetic resonance imaging; nr-axSpA, non-radiographic axial spondyloarthritis; BASRI, Bath Ankylosing Spondylitis Radiology Index; PsA, psoriatic arthritis; DXA, dual-energy X-ray absorptiometry; SLICC, Systemic Lupus International Collaborating Clinics; ESSDAI, EULAR Sjögren’s Syndrome Disease Activity Index.

## 4 Osteo-immunology in immune-mediated inflammatory diseases

The etio-pathogenesis of osteoporosis is multifactorial, with genetic, hormonal, dietary, and lifestyle factors, as well as concomitant medications and diseases, contributing to its development. Osteo-immunology also plays a fundamental role in pathogenesis.

### 4.1 Effect of immune-mediated inflammatory diseases on the regulation of bone remodeling by the immune system

**Figure 1:**
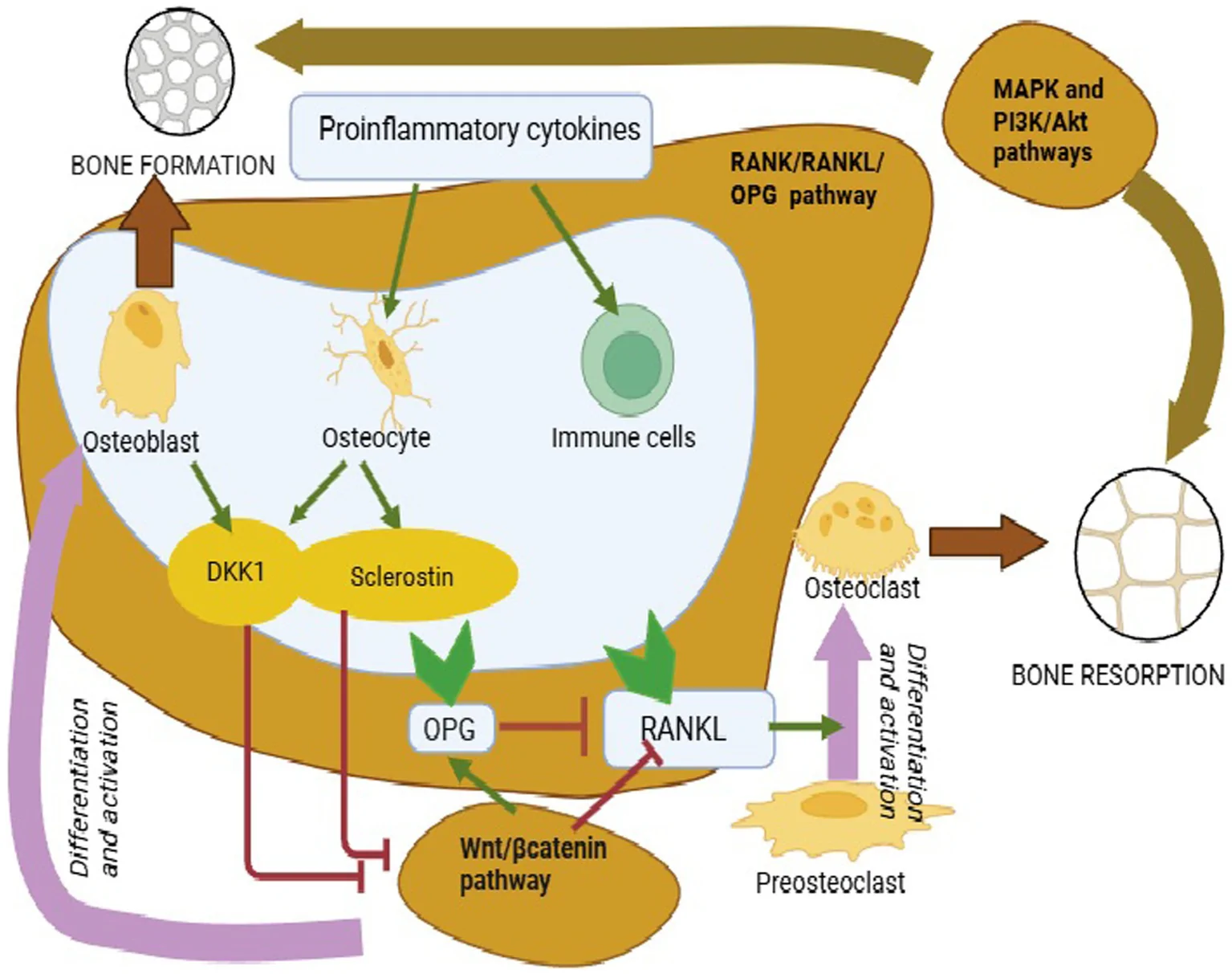
Regulation of bone metabolism under physiological conditions. Bone remodeling is regulated by various signaling pathways. The RANK/RANKL/OPG system controls osteoclast differentiation and bone resorption. Inflammatory cytokines, mainly TNF-α, IL-1, IL-6, and IL-17, promote RANKL expression in osteoblasts, osteocytes, and immune cells. Receptor activator of nuclear factor κB ligand (RANKL) binds to its receptor (receptor activator of nuclear factor κB [RANK]) on pre-osteoclasts, inducing their differentiation and maturation into active osteoclasts. These cytokines also stimulate the production of osteoprotegerin (OPG), a soluble decoy receptor that prevents RANKL from activating pre-osteoclasts. The Wnt/*β*-catenin pathway promotes osteoblast differentiation and activity while inhibiting bone resorption by increasing OPG and decreasing RANKL expression. Its endogenous inhibitors, sclerostin and Dickkopf-1 (DKK1), help maintain bone remodeling homeostasis by balancing bone formation and resorption. The PI3K/Akt and MAPK pathways act as dual regulators of bone osteoblast activity to maintain bone homeostasis.

The immune system has an important regulatory function in bone remodeling. Certain cytokines, such as TNF-*α*, IL-1, IL-6, and IL-17, promote osteoclast activation and differentiation by increasing the expression of receptor activator of nuclear factor κB ligand (RANKL) on osteoblasts and immune cells. RANKL binds to its receptor, RANK, on osteoclast precursors, stimulating their maturation and promoting bone resorption. Other cytokines also play an important role in bone metabolism. IL-7, IL-8, IL-11, IL-15, and IL-20 are osteoclastogenic, primarily promoting osteoclast differentiation and activity, whereas IL-3, IL-4, IL-10, IL-13, IL-18, IL-19, IL-27, IL-29, IL-32, IL-33, IL-37, and interferon (IFN) act as anti-osteoclastogenic cytokines, inhibiting osteoclastogenesis (33). Moreover, although the main source of osteoprotegerin (OPG) is osteoblasts, the immune system is also involved in its production, with B lymphocytes being the primary contributors. OPG is an endogenous inhibitor of RANKL, acting as a “decoy” receptor that prevents RANK activation and thus regulates bone resorption (see Figure 1).

**Figure 2:**
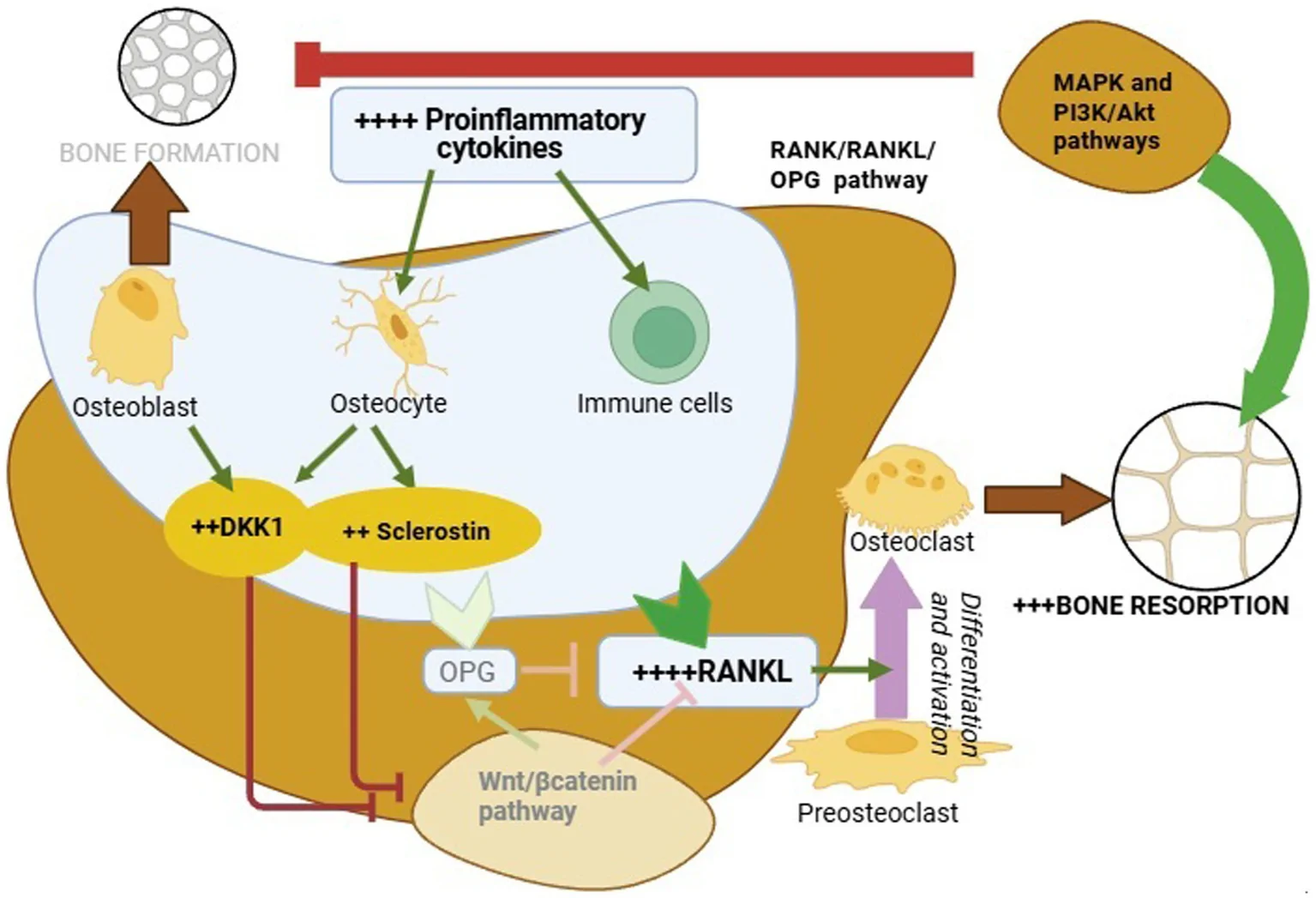
Regulation of bone metabolism under inflammatory conditions. Activated lymphocytes, macrophages, and dendritic cells secrete inflammatory cytokines, mainly TNF-α, IL-1, IL-6, and IL-17, which promote RANKL expression and decrease OPG production, leading to enhanced osteoclast activation. Sclerostin and DKK1 are upregulated under inflammatory conditions, inhibiting the Wnt/β-catenin pathway and further favoring osteoclast activation. During chronic inflammation, the MAPK and PI3K/Akt pathways disrupt the balance between bone formation and resorption, promoting osteoclast differentiation, activation, and survival.

IMIDs are characterized by chronic immune activation that not only affects the target organs of each disease but also disrupts bone tissue homeostasis. They can modify the normal regulatory function of the immune system in bone remodeling. Activated T lymphocytes, B lymphocytes, macrophages, and dendritic cells secrete inflammatory cytokines such as TNF-*α*, IL-1, IL-6, and IL-17, which promote bone resorption through the expression of RANKL and can induce massive osteoclast activation. In contrast, other cytokines, including IFN-*γ*, IL-4, and transforming growth factor-*β*, exert predominantly inhibitory effects on osteoclastogenesis. The balance between these pro- and anti-osteoclastogenic cytokines is likely to differ between disease states, potentially accounting for differences in predisposition to bone loss. On the other hand, TNF-*α*, IL-1, IL-6, and IL-17 can inhibit osteoblast activity in two ways: directly, by reducing their function or even inducing apoptosis in osteoblasts and their precursors, and indirectly, by increasing RANKL levels while decreasing OPG production (15, 33). Furthermore, TNF-*α* decreases the production of OPG, thereby favoring bone resorption (see Figure 2).

### 4.2 Immune-mediated inflammatory diseases and other pathways that modulate bone remodeling

Chronic immune activation may also interfere with bone tissue homeostasis through other pathways that modulate bone remodeling.

The Wnt/*β*-catenin pathway stimulates bone formation by promoting osteoblast differentiation and activity and inhibits bone resorption by reducing RANKL expression and increasing OPG levels (see Figure 1). One of the inhibitors of this pathway is sclerostin, which is elevated not only in inflammatory diseases but also in conditions of reduced mechanical load or immobility, thereby promoting bone resorption in IMIDs (13, 34) (see Figure 2). Another inhibitor of this pathway is Dickkopf-1 (DKK1), whose secretion is increased in IMIDs such as RA, SLE, and systemic sclerosis through inflammatory cytokines such as TNF-*α* and IL-6 (14) (see Figure 2). Immobility or decreased physical activity due to pain or functional limitation is also common in these diseases. Bone requires mechanical stress to maintain its function, and when mechanical load is lost, the balance between osteoblast activity and osteoclast activity is disturbed, favoring bone resorption (15). Furthermore, under conditions of reduced mobility, osteocytes increase the secretion of sclerostin, which inhibits the Wnt/*β*-catenin pathway, decreasing bone formation and promoting resorption (34) (see Figure 2).

The MAPK pathway is also activated by pro-inflammatory cytokines. This pathway acts as a dual regulator by promoting osteoclast formation via the p38/JNK pathway and stimulating osteoblast formation via the ERK1/2 pathway (see Figure 1). However, under conditions of inflammation or estrogen deficiency, this signaling balance shifts in favor of bone resorption (35) (see Figure 2).

TNF-*α*, IL-1, IL-6, and IL-17 also affect the PI3K/Akt pathway, which plays a dual role in bone: it protects and promotes bone formation by stimulating osteoblastic differentiation and survival, although it can also enhance osteoclast activity (see Figure 1). In chronic inflammatory conditions, this balance is disrupted, and TNF-*α* promotes the activation, differentiation, and survival of osteoclasts. In osteoblasts, its effect can be inhibitory, especially during prolonged inflammatory states, impairing bone formation and favoring osteoporosis (36, 37) (see Figure 2). Under inflammatory conditions, IL-6 activates PI3K/Akt in osteoclast precursors to promote cell survival and can also act in concert with other cytokines (TNF-α, IL-1), amplifying inflammation and contributing to bone loss (38, 39). Under physiological conditions, IL-17 activates PI3K/Akt in osteoblasts and mesenchymal stem cells, promoting osteogenic differentiation and mineralization; however, its effect on this pathway under inflammatory conditions remains unclear (40). Finally, the role of IL-1 in PI3K/Akt signaling under inflammatory conditions has not been fully characterized, although IL-1*α* directly activates this pathway in mature osteoclasts, promoting their survival under physiological conditions (41).

RA is a clear example of the importance of osteo-immunology in the pathogenesis of osteoporosis in IMIDs. Elevated levels of TNF-α, IL-1, and IL-6, together with chronic inflammation, promote bone resorption through the PI3K/Akt and MAPK pathways, increase RANKL expression, and decrease OPG secretion. Furthermore, serum levels of DKK-1 and sclerostin are elevated, inhibiting the Wnt/*β*-catenin pathway and impairing bone formation. Secretion of sclerostin can also be enhanced by reduced mechanical load. This is common in patients with pain or functional limitations, further contributing to bone loss (14).

The therapeutic approach to IMIDs includes bDMARDs and tsDMARDs, which act by blocking key cytokines involved in the pathophysiology of inflammation and bone metabolism. Consequently, in addition to controlling disease activity, these treatments may exert a beneficial effect on bone metabolism.

## 5 Effect of bDMARDS and tsDMARDS on bone metabolism in adults with immune-mediated inflammatory diseases

### 5.1 Methodology

As our aim was to identify the available evidence as comprehensively as possible, despite this being a narrative review, we conducted a search strategy to identify studies evaluating the effects of bDMARDs and tsDMARDs on bone mass in patients with IMIDs. The studies were identified using sensitive search strategies in the following key bibliographic databases: Ovid MEDLINE, Embase, and the Cochrane Central Register of Controlled Trials from 2015 to August 18, 2025. A date limit of 2015 was applied to narrow the search results because previous reviews had already included articles published before that year. Combinations of keywords, including MeSH terms and free-text terms, were used. An expert librarian reviewed the search strategies. Details of the strategies and the search terms are shown in Table 2.

**Table 2:** Search criteria.

Criteria	Description
Keywords (general terms)	- Osteoporosis [mesh]
	- Bone diseases, metabolic [mesh]
	- Osteopenia
	- Bone mass
	- Bone density
Keywords (drugs/treatments)	- Biological therapy [mesh]
	- Biological products [mesh]
	- Biological therapy
	- Molecular targeted therapy [mesh]
	- Targeted synthetic therapy
	- Targeted molecular therapy
	- Anti-TNFα agents: infliximab, adalimumab, golimumab, certolizumab, etanercept
	- IL-6 receptor inhibitors: tocilizumab, sarilumab
	- IL-23 inhibitors: ustekinumab, guselkumab, risankizumab
	- IL-17 inhibitors: secukinumab, ixekizumab, bimekizumab
	- JAK inhibitors: tofacitinib, baricitinib, upadacitinib, filgotinib
	- Other immunomodulators: rituximab, abatacept, belimumab, anifrolumab, anakinra, apremilast

The studies retrieved by the strategies mentioned were included if they met the following pre-established criteria:

1. The population comprised adult patients diagnosed with IMIDs (RA, axSpA, PsA, psoriasis, Sjögren’s syndrome, systemic sclerosis, inflammatory myopathies, systemic vasculitis, Behçet’s disease, multiple sclerosis, and IBD [Crohn’s disease and ulcerative colitis]).
2. The intervention was treatment with a bDMARD and tsDMARD.
3. No comparator was included.
4. The outcomes were bone mass, osteoporosis, and fractures.

Finally, a manual search was performed by reviewing all the references of the included studies. Each reference list was screened to identify additional studies that met the predefined inclusion criteria, and potentially relevant titles were evaluated by reviewing the abstract or, when necessary, the full text to determine eligibility.

Studies not published in Spanish or English and those for which the full text was not available were excluded. Narrative reviews were also excluded.

In total, 530 references were included (529 in English and 1 in Spanish). From the initial search, 41 articles were selected based on their titles and abstracts and were subsequently examined in full. Moreover, given its importance, one article was included through the manual search.

Table 3 provides a summary of the effect of bDMARDs and tsDMARDs on bone mass and the incidence of fracture.

**Table 3:** Treatments by class and effects on bone mass and fracture incidence.

Drug class	Type of drug (bDMARD, tsDMARD)	General effect on bone mass	Effect on fracture incidence
Anti-TNF	bDMARD	Stabilization or increase	No effect
Tocilizumab (IL-6 receptor Inhibitor)	bDMARD	Stabilization or increase	No effect
Rituximab (CD20 inhibitor)	bDMARD	Mixed data, possible benefit	No effect
Abatacept (selective T-cell co-stimulation inhibitor)	bDMARD	Stabilization or increase	No effect
Secukinumab (IL-17 inhibitor)	bDMARD	Possible increase (single study)	No data
JAK inhibitors	tsDMARD	Stabilization or increase	No effect
IL-23 inhibitors	bDMARD	No data	No data
Belimumab	bDMARD	No data	No data
Anifrolumab	bDMARD	No data	No data
Apremilast (PDE4 inhibitor)	tsDMARD	No data	No data

bDMARD, biologic disease-modifying antirheumatic drug; tsDMARD, targeted synthetic disease-modifying antirheumatic drug; IL, interleukin; PDE4, phosphodiesterase 4.

### 5.2 TNF-*α* inhibitors

TNF-α inhibitors (TNFi) are widely used in the management of various IMIDs, such as RA, axSpA, PsA, and IBD. In this context, TNFi have not only demonstrated efficacy in controlling inflammation but may also play a beneficial role in preserving bone metabolism.

#### 5.2.1 Effect on bone mineral density in rheumatoid arthritis and in axial spondyloarthritis

Su et al. (42) conducted a retrospective analysis of 3,509 Taiwanese patients with RA aged >50 years and without osteoporosis. They found that 37% of patients developed osteoporosis, with a lower crude incidence among those treated with TNFi (etanercept, adalimumab, certolizumab, and golimumab) (40.4 events per 1,000 person-years [PYs] [95% CI: 28.6–52.2]) and other bDMARDs (tofacitinib, baricitinib, and tocilizumab) (40.1 events per 1,000 PYs [95% CI: 8.0–72.1)] than in the combination group abatacept/rituximab (48.6 events per 1,000 PYs [95% CI: 14.9–82.3]) and the conventional synthetic DMARD (csDMARD) and untreated groups. In addition, TNFi were more protective against osteoporosis than no treatment (HR 0.69, 95% CI: 0.50–0.96).

In 2019, Ashany et al. (43) published a systematic review of the literature on the effects of any available TNFi (adalimumab, etanercept, infliximab, certolizumab, and golimumab) on bone mass and fracture risk in individuals aged 18 years or older with axSpA, with a follow-up of 2–4 years. The authors analyzed 27 studies and observed increases in BMD in the lumbar spine (LS) (3.2–14.9%) and in the hip (2.26–4.7%) without reducing vertebral fractures. The effect was attributed to the use of TNFi as a class; no comparisons were made between the different TNFi.

Other published prospective studies, albeit with smaller patient cohorts, report similar findings, showing stabilization or improvement in BMD following TNFi treatment, with greater gains observed in the LS than in the femoral neck (FN). These results were obtained in patients with RA and ankylosing spondylitis using the TNFi most employed in clinical practice (infliximab, adalimumab, etanercept, certolizumab, and golimumab) (44–48). Analyses were generally performed using the TNFi group (which could include several of these agents), except for one study in RA patients that assessed adalimumab only (44) and another study in ankylosing spondylitis patients that evaluated etanercept only (46). In most studies, IMID was active at baseline, and patients were biologic-naïve. Follow-up periods ranged between 1 and 4 years. In most studies, there was no comparator group, except one single-center study comparing RA patients treated with adalimumab versus methotrexate, which showed favorable results for adalimumab (44), and another multi-center study comparing RA patients treated with TNFi versus csDMARDs versus abatacept, which demonstrated a significant and positive difference in the percentage change in BMD in FN, total hip (TH), and LS with abatacept compared with the other treatments in an adjusted regression analysis (47). Another single-center study evaluated patients with ankylosing spondylitis and low bone mass over 1 year of TNFi therapy, finding a significant increase in BMD at the LS and FN compared with untreated controls (48).

When assessing the prolonged effect of TNFi on BMD, the study by Durnez et al. (49), published prior to the predefined search period, deserves special mention owing to the long follow-up period and the inclusion of a control group not taking TNFi. In their single-center retrospective study, the authors analyzed patients with axSpA treated with TNFi for at least 4 years, with a mean follow-up of 6.5 years. They measured changes in BMD after a minimum of 4 years of treatment and compared the results with those of untreated controls. A significant mean increase of 11.8% (±12.8%) was observed in the LS and 3.6% (±9.3%) in the greater trochanter, with no significant changes in TH BMD in the TNFi group. Increases in BMD were significantly greater in the TNFi group at the LS, FN, and trochanter (while no significant difference was observed for TH). These findings support the notion that prolonged TNFi therapy significantly increases BMD.

The available studies suggest that TNFi preserve or improve BMD in RA and axSpA, although the magnitude and skeletal distribution of these effects are variable (42–49). Improvements were more consistently observed at the LS than at the hip, with large population-based data in RA suggesting that TNFi prevent osteoporosis (42). Variability across studies is explained by differences in design, sample size, follow-up duration, disease stage, baseline BMD, inflammatory burden, and comparator groups, introducing residual confusion. In axSpA, interpretation of lumbar BMD is further complicated by syndesmophytes and structural spinal changes, which may overestimate bone densitometry measurements despite attempts to account for this bias (49). Collectively, the predominance of observational studies and limited controlled comparisons restrict causal inference. Despite these limitations, the consistency of findings across independent cohorts supports a genuine effect on bone mass. Taken together, it can be reasonably considered that the overall quality of the evidence is low to moderate.

#### 5.2.2 Effect on bone turnover markers

The behavior of BTMs following TNFi treatment has also been investigated. BTMs reflect the dynamic activity of bone, specifically the processes of bone formation and resorption, and are useful for assessing bone metabolism in various diseases and for monitoring the response to treatments that impact bone health.

##### 5.2.2.1 Rheumatoid arthritis and spondyloarthritis

TNFi treatment has been associated with changes in bone formation markers (P1NP, osteocalcin, and an increased P1NP/CTX ratio) as well as in bone resorption (CTX). In patients with active RA treated with adalimumab for 1 year, a decrease in CTX was observed compared to patients receiving methotrexate, while no significant differences were found in P1NP (44). In another cohort of 36 patients with RA and 17 with active axSpA treated with etanercept or certolizumab for 1 year, increases in P1NP and the P1NP/CTX ratio and stabilization of CTX were reported (45). Jura-Półtorak et al. (50) compared the behavior of BTMs in RA patients and found significantly lower P1NP and higher CTX levels than in healthy controls; however, after 15 months of TNFi therapy combined with methotrexate, P1NP increased significantly, and CTX-I decreased significantly relative to baseline. In their study of patients with ankylosing spondylitis and low bone mass, Li et al. (48) observed a significant decrease in CTX levels compared to baseline and an increase in bone-specific alkaline phosphatase and P1NP.

Nevertheless, previous results are not entirely consistent across studies, as a prospective study including 31 patients with RA, PsA, and axSpA treated with TNFi for 1 year did not show significant changes in BTMs, although a significant reduction in sRANKL levels and in the sRANKL/OPG ratio was observed, indicating diminished osteoclastogenic signaling (51).

##### 5.2.2.2 Inflammatory bowel disease

Small-scale studies in patients with IBD have also investigated BTMs, reporting lower baseline osteocalcin than controls, followed by a significant and early increase in osteocalcin and P1NP and a non-significant decrease in CTX (52, 53).

The studies suggest that TNFi shift bone remodeling toward a more favorable balance by reducing bone resorption and enhancing bone formation, although the magnitude of these effects varies across IMIDs and biomarkers assessed (44, 45, 48, 50–53). Decreases in CTX together with increases in P1NP, osteocalcin, or bone-specific alkaline phosphatase were more consistently observed in prospective disease-specific cohorts (44, 45, 48, 50, 52, 53), whereas no significant changes in conventional BTMs were detected in the small heterogeneous cohort reported by Aguilar Del Rey et al. (51), despite significant reductions in sRANKL and the sRANKL/OPG ratio. These discrepancies are likely explained by differences in sample size, study design, disease heterogeneity, comparator groups, follow-up duration, concomitant therapies, and baseline inflammatory burden, all of which may influence the sensitivity of BTMs to detect treatment effects. In summary, the overall quality of the evidence can be considered low, with generally consistent findings but limited by small observational cohorts and methodological heterogeneity.

#### 5.2.3 Effect on fracture risk

##### 5.2.3.1 Rheumatoid arthritis

We identified three relevant studies on the risk of fracture in RA. In their systematic review and meta-analysis on the impact of bDMARDs on risk of any type of fracture in patients with RA, Shao et al. (54) found no significant differences in fracture risk between patients treated with TNFi (adalimumab, etanercept, infliximab, certolizumab pegol, and golimumab) and those treated with csDMARDs and/or corticosteroids and/or non-steroidal anti-inflammatory drugs (OR 0.96 [95% CI: 0.75–1.23, *p* = 0.77]).

In a retrospective population-based study including 5,357 patients followed for a mean of 1.2 years, no differences were found in the risk of osteoporotic fractures when TNFi were compared with abatacept (HR 0.91; 95% CI: 0.48–1.71) or with tocilizumab (HR 1.00; 95% CI: 0.55–1.83). Similar results were observed for both vertebral and non-vertebral fractures (55).

Regarding non-vertebral fractures, a retrospective multi-center study of a large cohort found no significant differences in the risk of non-vertebral fractures (hip, humerus, pelvis, and wrist) between various bDMARDs and tsDMARDs (abatacept, certolizumab, etanercept, golimumab, infliximab, abatacept, rituximab, tocilizumab, and tofacitinib) and adalimumab in patients with RA. Classical risk factors (age, frailty, glucocorticoid use, and osteoporosis) were associated with a higher absolute risk of non-vertebral fractures, although they did not modify the relative risk between different treatments, which remained similar across all subgroups (56).

##### 5.2.3.2 Axial spondyloarthritis

As for patients with axSpA, the results of a systematic review (see above) show that the gains in BMD observed following TNFi treatment do not appear to be consistently associated with a reduction in fracture risk (43). This apparent paradox could be explained in part by the fact that fractures were a secondary outcome in the review and the number of articles that assessed them was small, but, furthermore, lumbar spine BMD may be influenced by syndesmophytes and ligament ossification, while the risk of fracture is also determined by bone quality, trabecular microarchitecture, structural vertebral damage and alterations in spinal biomechanics, which were not analyzed in the review. Maas et al. (57) analyzed 105 patients with active ankylosing spondylitis at baseline and after 4 years of TNFi (infliximab, etanercept, and adalimumab). Despite significant improvements in BMD and clinical disease activity, 20% of patients developed new vertebral fractures. This single-arm study showed that the variables associated with the development of new fractures were older age, longer duration of smoking, poorer physical function (Bath Ankylosing Spondylitis Functional Index), lower lumbar BMD (*Z*-score ≤−2), presence of previous vertebral fractures, and use of anti-osteoporotic treatment at baseline.

Beek et al. (58) reported comparable findings in their cohort of 135 patients with active ankylosing spondylitis treated with TNFi (infliximab, etanercept, and adalimumab) for 4 years, observing significant increases in BMD. However, 19.6% of patients developed new vertebral fractures. The authors also found that age, low baseline BMD (particularly in the LS), and greater baseline radiographic damage (Modified Stoke Ankylosing Spondylitis Spinal Score) were associated with new vertebral fractures, suggesting that factors other than low bone mass contribute to fracture risk.

Similar results were obtained by van der Weijden et al. (46) in their prospective cohort of 49 biologic-naïve patients with active ankylosing spondylitis treated with etanercept for 2 years: despite significant increases in BMD, particularly in the LS, the number of vertebral fractures doubled following treatment. The study did not identify any clinical or laboratory variables associated with the development of new vertebral fractures.

The available evidence suggests that improvements in BMD and BTMs induced by TNFi do not translate into a reduced fracture risk across IMIDs (43, 46, 54–58). In RA, a meta-analysis and two large active-comparator observational studies (csDMARDs or other biologic and targeted synthetic therapies), which provide moderate-quality evidence, found no differences in fracture risk between TNFi and other treatment strategies (54–56), although residual confounding by disease severity, glucocorticoid exposure, and baseline osteoporosis, together with heterogeneous fracture definitions and relatively short follow-up, may have obscured modest treatment effects. In contrast, in axSpA, new vertebral fractures were identified despite significant increases in BMD (46–58), indicating that structural spinal damage, impaired bone quality, biomechanical vulnerability, and pre-existing fractures contribute to fracture risk beyond bone mass alone; furthermore, the combination of small prospective cohorts, limited follow-up, and the absence of active comparators results in low-quality evidence.

#### 5.2.4 Expert opinion

The skeletal benefits of TNFi therapy are likely to be due to a combined effect of direct modulation of bone metabolism and the control of systemic inflammation, as well as associated improvements in physical function, mobility, and reduced glucocorticoid exposure.

In patients with RA, increased BMD and modulation of BTMs could maintain fracture risk at a similar level to that of patients with less active disease who do not require biologic therapy. Moreover, the fracture risk appears to be comparable between TNFi agents and other biologics.

Patients with ankylosing spondylitis continue to experience vertebral fractures associated with classic risk factors, as well as disease-specific factors, such as radiographic damage and bone quality, despite the increases in bone mass induced by TNFi therapy. However, the study designs prevent us from determining whether the fracture risk is similar to that of patients not receiving biologics or treated with other biologics.

### 5.3 IL-6 receptor inhibitors

IL-6 promotes bone resorption through the expression of RANKL, enhancing osteoclast differentiation and activation. Under inflammatory conditions, IL-6 also stimulates the production of other cytokines such as TNF-*α* and IL-1β, further amplifying bone resorption.

#### 5.3.1 Effect on bone mineral density in rheumatoid arthritis

Several observational studies have evaluated the effect of tocilizumab on BMD in patients with active RA in whom csDMARDs and/or bDMARDs or tsDMARDs have proven ineffective. Two prospective multi-center studies evaluated BMD, finding that it remained stable after 1 or 2 years of follow-up (59, 60). However, this outcome was secondary, and in one of the studies, the results were not adjusted for the use of anti-osteoporotic treatment (60). Al Khayyat et al. (61) analyzed 18 patients with RA treated with tocilizumab for 12 months, observing a significant increase in LS BMD, while no significant changes were detected in the BMD of the FN and TH. Chen et al. (62) conducted a prospective observational study involving 76 RA patients. After 2 years of treatment with tocilizumab, changes in BMD were compared according to anti–cyclic citrullinated peptide (ACPA) positivity and glucocorticoid dose. ACPA-positive patients had significantly lower baseline T-scores in LS and FN. After 2 years, a significant increase in FN BMD was observed in the ACPA-positive subgroup and in those receiving low doses of glucocorticoids (5 mg/day), whereas no relevant changes were detected in ACPA-negative patients. This could be partly explained by the fact that some of the ACPA-negative cases were not true RA (the report does not specify how many were RF-positive) and that IL-6 inhibition did not have the expected effect. Furthermore, it has been suggested that ACPA may play a potential role in bone loss by contributing to osteoclastogenesis and bone resorption (cite reference); therefore, ACPA-positive RA patients may derive greater benefit from IL-6 inhibition due to a more pronounced involvement of this pathway.

Overall, the available studies suggest that IL-6 receptor inhibition prevents systemic bone loss in RA, with a low to moderate quality of evidence (59–62). The larger prospective multi-center cohorts reported stable BMD over 1–2 years (59, 60), while significant increases were mainly observed in smaller or selected populations, including patients with baseline osteopenia, ACPA positivity or lower glucocorticoid exposure (61, 62). These differences are likely explained by population heterogeneity, baseline fracture risk and concomitant treatments rather than by conflicting treatment effects. The absence of control groups, the fact that BMD was a secondary outcome, and potential confounding from previous biologic or anti-osteoporotic therapies further limit causal interpretation (59–62).

#### 5.3.2 Effect on bone turnover markers

##### 5.3.2.1 Rheumatoid arthritis

Results on the effect of tocilizumab on BTMs show some heterogeneity across studies in RA patients. A decrease in CTX levels has been observed after 2 years of treatment, especially in ACPA-positive patients (62). In the study by Briot et al. (59), significant increases in P1NP and in the P1NP/CTX ratio were reported, although these changes did not correlate with variations in BMD. Additionally, the authors observed a significant reduction in serum DKK-1 concentrations, as suggested elsewhere (61). DKK-1 is a protein that is highly elevated in active RA and acts as an inhibitor of the Wnt signaling pathway. The reduction in DKK-1 levels could therefore alleviate Wnt-mediated osteoblastic inhibition, promoting bone formation. However, the analysis of bone remodeling in this study was a secondary objective.

##### 5.3.2.2 Polymyalgia rheumatica

A prospective study involving 20 patients with early-onset polymyalgia rheumatica treated with tocilizumab—without glucocorticoids during the first 12 weeks—showed a consistently significant increase in P1NP and an improvement in the coupling between bone formation and resorption, suggesting a recovery of bone turnover balance mediated by inhibition of IL-6 (63).

Overall, the available evidence suggests that IL-6 receptor inhibition shifts bone turnover toward a more favorable remodeling profile, with a low to moderate quality of evidence (59, 61–63). Reductions in bone resorption markers and in DKK-1, together with increases in P1NP or the P1NP/CTX ratio, support a restoration of the bone formation–resorption balance, while differences in individual BTMs are likely related to variations in study design, follow-up duration, sample size and patient characteristics, including ACPA status and glucocorticoid exposure (59, 61–63). The absence of control groups and the use of BTMs as secondary or surrogate outcomes limit causal inference. In PMR, the glucocorticoid-free induction phase supports the attribution of these effects to IL-6 blockade, although the evidence remains limited to a small uncontrolled study (63).

#### 5.3.3 Effect on fracture risk in rheumatoid arthritis

As previously discussed in the section on TNFi, two published studies compared the risk of fracture in patients with RA initiating TNFi therapy with the risk in those starting with other bDMARDs, including tocilizumab. No statistically significant differences were observed between the different treatment groups in either study, suggesting a comparable fracture risk across these classes of drugs (55, 56), with moderate-quality evidence. The concordant findings from two large real-world comparative cohorts strengthen the consistency of the evidence, although both studies were observational and fractures were assessed as secondary safety outcomes rather than primary endpoints. Despite the use of propensity score methods and multivariable adjustment, residual confounding related to disease severity, cumulative glucocorticoid exposure, baseline bone health and other unmeasured fracture risk factors cannot be excluded. Moreover, comparisons were restricted to biologic therapies, precluding conclusions on the absolute anti-fracture effect of IL-6 blockade.

#### 5.3.4 Expert opinion

Inhibition of the IL-6 receptor with tocilizumab may have a beneficial effect on bone metabolism in patients with IMIDs, particularly in RA, by stabilizing or modestly improving BMD and positively influencing BTMs. This biological profile is consistent with the suppression of IL-6-mediated osteoclastic activation and the restoration of the balance between bone formation and resorption, which together may translate into an impact on fracture risk comparable to that observed with TNF inhibitors in comparative studies. However, the limited number of studies and the fact that bone outcomes have been assessed as secondary endpoints prevent definitive conclusions from being drawn regarding the skeletal benefits of IL-6 inhibition.

### 5.4 CD20 inhibitors (rituximab)

CD20 is a surface protein expressed on B lymphocytes. B cells play a crucial role in bone homeostasis by producing RANKL and its physiological inhibitor OPG. However, under chronic inflammatory conditions such as RA, B cells produce more RANKL—mainly from activated T cells, but also from activated B cells—and insufficient OPG to counterbalance it. This shifts the RANKL/OPG ratio in favor of bone resorption and leads to decreased BMD (64).

#### 5.4.1 Effect on bone mineral density and bone turnover markers in rheumatoid arthritis

Studies on BMD and bone remodeling focus on patients with RA who exhibit an inadequate response to csDMARDs and/or other biologic agents (65–67). LS BMD and stability in the FN after 18 months of treatment increased significantly in a retrospective cohort of 20 postmenopausal women with RA (65). Lim et al. (66) prospectively evaluated 20 seropositive RA patients at 12 months: 12 received a single course of rituximab, while eight required a second course owing to persistent disease activity. In the group receiving a single course, BMD remained stable, RANKL levels decreased, and the OPG/RANKL ratio increased, correlating with reduced disease activity. However, in the group needing two courses, TH BMD decreased significantly, whereas RANKL levels and disease activity increased. Inflammatory activity measured by DAS28 was associated with bone loss. CD19 levels decreased markedly in both groups, indicating successful B-cell depletion. Nevertheless, this alone was insufficient to prevent bone loss in the absence of adequate control of inflammation, suggesting that persistent RA activity is a key driver of BMD loss in these patients. Wheater et al. (67) reported results from a prospective cohort of 36 RA patients: after 1 year of rituximab treatment, no changes in BMD were observed at the LS or distal radius, although significant losses were noted at the FN and TH. Of note, the measurements recorded were secondary outcomes. Interestingly, P1NP levels increased significantly without corresponding changes in resorption markers or DKK-1 levels. Overall, levels of inflammatory markers and disease activity scores decreased significantly at 12 months, indicating that although rituximab effectively controlled inflammation, this was not sufficient to prevent BMD loss at the hip.

Overall, the available evidence, although of low quality, suggests that the skeletal effects of rituximab depend largely on the degree of inflammation control rather than on B-cell depletion alone (65–67). While LS BMD was maintained or improved in some cohorts, hip BMD declined in patients with persistent disease activity or inadequate treatment response (66, 67). These differences are likely explained by variations in patient characteristics, including studies enrolling only women (65) or only seropositive patients (66), disease control and concomitant therapies, including the use of anti-osteoporotic treatment in one study (65), as well as by the small sample sizes and uncontrolled design across all cohorts. Changes in BTMs, particularly the reduction in RANKL and increase in the OPG/RANKL ratio in clinical responders, further support the link between effective inflammation suppression and preservation of bone health (66).

#### 5.4.2 Effect on fracture risk in rheumatoid arthritis

Regarding the efficacy of reducing fracture risk, we were unable to find a study that examined this outcome by specifically evaluating the effect of rituximab. Su et al. (42) analyzed 3,509 patients with RA receiving various csDMARDs and bsDMARDs and found a higher crude incidence of fractures in the group that could be treated with rituximab or abatacept than in the other treatment groups, with a higher fracture risk than the group treated with methotrexate plus other csDMARDs (HR 5.88, 95% CI: 2.07–16.66; *p* = 0.001). Interestingly, this occurred despite no observed increase in the incidence of osteoporosis. The authors concluded that the higher fracture risk observed in this group likely reflects greater disease severity and increased cumulative exposure to risk factors, rather than a direct detrimental effect of these agents on bone. Moreover, they recommended interpreting these findings with caution and validating them in future studies with larger sample sizes and more rigorous adjustment for confounding variables. Therefore, this study does not enable us to determine the individual effect of either drug on fracture risk.

#### 5.4.3 Expert opinion

The available data suggest that preservation of bone health is primarily associated with effective suppression of inflammatory disease activity rather than with B-cell depletion itself. Consequently, no firm conclusions can be drawn regarding the direct effect of rituximab on BMD or bone metabolism.

### 5.5 Abatacept

Abatacept is a biologic agent used in the treatment of RA and PsA that acts by inhibiting T-cell co-stimulation. In murine models, abatacept has been shown to induce anergic T cells to produce Wnt-10b, a protein that activates the Wnt signaling pathway in osteoprogenitor cells, thereby stimulating osteoblast differentiation and activity and promoting bone formation. Abatacept can also increase the expression of sclerostin, a protein that inhibits bone formation and promotes bone resorption. In individuals with normal T-cell function, the anabolic effect of Wnt-10b is thought to predominate, supporting bone mass preservation or gain. However, in immunocompromised individuals or patients with T-cell deficiencies, reduced Wnt-10b production may enable the inhibitory effects of sclerostin to prevail, potentially leading to bone loss (68).

#### 5.5.1 Effect on bone mineral density and bone turnover markers in rheumatoid arthritis

Furthermore, studies on BMD and BTMs focused on patients with RA refractory to csDMARDs and/or bDMARDs or tsDMARDs. The AIRTIGHT study, a prospective comparative non-randomized study comparing the effects of abatacept and other bsDMARDs (77.4% TNFi and 22.6% tocilizumab) on bone metabolism at 1 year in 165 RA patients, reported that the percentage change in BMD in the FN was greater in the abatacept group (+0.97%) than in the non-abatacept group (−2.19%; *p* = 0.026). No differences were observed in LS BMD or BTMs between groups. The use of abatacept was associated with an increase in BMD in the FN (OR 8.84, 95% CI: 1.08–72.4) (69).

A multicentre, prospective observational study including 485 RA patients with a 3-year follow-up found that in the abatacept group, BMD remained stable at all measurement sites (LS, FN, and TH), whereas in the csDMARD and TNFi groups there was a significant decrease in BMD at some sites. Abatacept conferred superior protective effects on BMD compared to the other groups because, in terms of regression-adjusted percent change in BMD, a significant difference was observed at all measured sites in favor of abatacept. Anti-osteoporotic therapy had a protective effect in all groups (47).

Regarding BTMs, a prospective single-center study conducted in 33 patients with seropositive RA who were starting abatacept showed increases in bone formation markers (P1NP and B-ALP) at 6 and 12 months, with no significant changes in resorption markers (70).

The available evidence, which is of moderate quality, suggests that abatacept preserves BMD and promotes a pattern of bone remodeling that favors bone formation, although the extent of these effects varies between studies (47, 69, 70). The greater protection observed in the largest cohort with the longest follow-up (47), compared with the more modest FN benefit reported after 12 months in the AIRTIGHT study (69), is likely explained by differences in follow-up duration, comparator groups and statistical power. The increase in bone formation markers without changes in resorption markers is biologically consistent with the preservation of BMD but derives from a small uncontrolled cohort (70). As all studies were observational, residual confounding related to disease activity, prior biologic exposure and anti-osteoporotic treatment cannot be excluded.

#### 5.5.2 Effect on fracture risk in rheumatoid arthritis

The study by Su et al. (42) showed that the combined rituximab/abatacept group had a higher crude incidence of fractures in RA patients than other treatment groups and a higher risk of fracture than the methotrexate + other csDMARD group. However, it did not analyze the specific effect of abatacept or rituximab separately. In addition, the authors recommended interpreting these results with caution, suggesting validation and adjustment for confounding variables. In fact, two large comparative real-world studies found no significant differences in fracture risk between abatacept and TNF inhibitors after adjustment for measured confounders (55, 56), thus highlighting inconsistency in the current evidence.

#### 5.5.3 Expert opinion

Abatacept may have beneficial effects on bone health in patients with RA, with studies showing preservation of BMD and favorable changes in BTMs; however, the available evidence on fracture risk remains limited and shows some inconsistency.

### 5.6 Interleukin-17 inhibitors

IL-17 plays a key role in inflammatory processes and in regulating the balance between bone formation and resorption. This cytokine promotes osteoclast activation and inhibits the Wnt signaling pathway in osteoblasts, thereby reducing the capacity of these cells to form new bone tissue. Consequently, inhibition of IL-17 may potentially reverse such deleterious effects on bone mass (33).

#### 5.6.1 Effect on bone mineral density and bone turnover markers in ankylosing spondylitis

To date, only one published study has specifically evaluated the effect of IL-17 inhibition on bone mass. Braun et al. (71) conducted a *post hoc* analysis of the phase 3 MEASURE 1 trial, which included 104 patients with active ankylosing spondylitis treated with the IL-17 inhibitor secukinumab 150 mg every 4 weeks and followed for 2 years. The results demonstrated a significant increase in LS BMD (+4.7%). In contrast, only minimal changes were observed in TH and FN BMD. No significant radiographic progression was observed during the follow-up period, and no correlation was observed between the increase in lumbar BMD and radiographic progression, indicating that the stabilization or improvement in BMD did not coincide with progression of structural damage or formation of syndesmophytes. Additionally, no significant changes in BTMs were reported.

Data on the incidence of fracture were not available in this study.

#### 5.6.2 Expert opinion

Although the increase in lumbar BMD supports a possible beneficial effect of IL-17 inhibition, the absence of significant changes at hip sites or in BTMs suggests a modest or site-specific skeletal effect. As bone outcomes were exploratory and fracture risk was not assessed, the findings should be interpreted with caution. Further studies are required to confirm the impact of IL-17 inhibitors on bone health.

### 5.7 JAK inhibitors

The JAK–STAT pathway (Janus kinase–signal transducer and activator of transcription) is one of the main intracellular signaling routes activated by numerous cytokines, hormones, and growth factors that regulate inflammation, immunity, hematopoiesis, and bone metabolism. In chronic inflammatory states, cytokines such as IL-6, IL-1β, IFN-*γ*, and granulocyte-macrophage colony-stimulating factor activate the JAK–STAT pathway and promote osteoclast differentiation via RANKL. Activation of the JAK–STAT pathway by inflammatory cytokines increases the expression of Wnt inhibitors (DKK-1 and sclerostin), thereby inhibiting osteoblastic action and contributing to bone mass loss (72). However, animal studies showed that by inhibiting the JAK–STAT pathway with JAK inhibitors (JAKi), osteoclast differentiation could theoretically be suppressed by inhibiting RANKL expression, osteoblast function could be restored through the Wnt pathway, and systemic inflammation that perpetuates bone damage could be reduced, potentially improving bone mass (73–76).

#### 5.7.1 Effect on bone mineral density and bone turnover markers in rheumatoid arthritis

The available data on humans remain limited and are focused exclusively on patients with RA refractory to bDMARDs or tsDMARDs. In total, four prospective studies including samples of 30–46 patients followed for 12-13 months showed that treatment with tofacitinib and baricitinib stabilized or improved both areal and volumetric BMD (77–80). In addition, baricitinib was associated with improvements in bone biomechanics (78). These findings were generally accompanied by reduced disease activity (77, 78, 80).

Regarding bone remodeling, Hamar et al. (77) observed an increase in bone formation markers (osteocalcin, OPG), an increase in vitamin D3, and a decrease in resorption markers (CTX), suggesting a positive effect of tofacitinib on bone formation. In contrast, Adami et al. (79) reported an increase in sclerostin (a bone formation inhibitor) and a decrease in P1NP and bone-specific alkaline phosphatase (bone formation markers), albeit with no significant changes in BMD. Meanwhile, Schulz et al. (80) observed a reduction in alkaline phosphatase following treatment with baricitinib, indicating reduced bone remodeling.

The discrepancies between these studies reflect the complexity of bone remodeling in RA and the influence of multiple factors, including the type of marker assessed, the drug used, clinical response, and patient characteristics. A reduction in bone formation markers may reflect normalization of bone remodeling in patients with active inflammation, in whom remodeling is accelerated and imbalanced. Therefore, a decrease in these markers is not necessarily negative, as it may indicate disease control and bone stabilization. Accordingly, results should be interpreted in conjunction with clinical progress, BMD measurements, and the patient’s overall context.

In a retrospective study, Chen et al. (81) compared 363 patients with RA treated with csDMARDs, TNFi, non-TNFi biologics (abatacept, rituximab, and tocilizumab), and JAKi (tofacitinib or baricitinib), finding that BMD remained stable at all major measured sites only in the JAKi group. In the other treatment groups, BMD and/or T-scores declined significantly. Moreover, JAKi were independently associated with an increase in FN BMD in ACPA-positive patients after adjustment for age, glucocorticoid use, interval between densitometry studies, and use of anti-osteoporotic medications.

Overall, the available evidence, which is of low to moderate quality, suggests that JAKi are associated with stabilization or a modest improvement in BMD in RA, although the findings regarding BTMs are heterogeneous (77–81). Prospective uncontrolled studies with tofacitinib and baricitinib (77, 78, 80) consistently report stable areal and volumetric BMD, with improvements in bone biomechanics in some analyses, whereas the larger comparative cohort study by Chen et al. (81) provides stronger evidence of BMD preservation compared with csDMARDs and biologics after multivariable adjustment. In contrast, discrepancies in BTMs (increased formation markers (osteocalcin and OPG) vs. reductions in P1NP and alkaline phosphatase) likely reflect differences in baseline inflammatory burden, marker selection, and interpretation of “normalization” of bone turnover under effective disease control (77, 79, 80). Limitations include small sample sizes, short follow-up, and the predominance of uncontrolled designs.

#### 5.7.2 Effect on fracture risk

In a *post hoc* analysis of pooled data from clinical trials of tofacitinib in RA, the risk of osteoporotic fracture was higher in patients treated with high-dose tofacitinib (10 mg twice daily) than in those treated with TNFi, according to data from the ORAL Surveillance study (P123LTE: integrated data from phase I, II, III studies and long-term extension [LTE] studies; phase III placebo-controlled trials; ORAL Surveillance: a phase IIIb/IV, open-label trial of tofacitinib [5 mg and 10 mg BID]) versus TNFi in patients aged ≥50 years with at least one cardiovascular risk factor) (HR for tofacitinib 10 mg vs. TNFi: 1.60; 95% CI: 1.09–2.36). However, only classic risk factors for fracture and ACPA positivity were associated with osteoporotic fracture risk. No treatment, including tofacitinib or TNFi, was independently associated with fracture risk after adjustment for these factors (82).

Pawar et al. (56) reported no significant differences in the risk of non-vertebral fractures between tofacitinib and adalimumab.

Therefore, the evidence regarding the risk of fracture associated with JAKi, as reported in studies of low to moderate quality, is inconsistent and appears to be explained primarily by patients’ baseline risk profiles rather than by the effect of the drug.

#### 5.7.3 Expert opinion

JAK inhibitors may have a neutral or beneficial effect on bone health in RA, leading to stabilization or modest improvements in BMD. However, the heterogeneity observed in the BTMs—likely related to differences in baseline inflammation and to the normalization of bone remodeling under clinical management—together with the inconsistent effects on fracture risk, limits a definitive interpretation of their skeletal impact.

### 5.8 Other drugs

No data are currently available regarding the effects of IL-23 inhibitors, belimumab, or anifrolumab on bone mass. As for IL-1 inhibitors, the available evidence is limited to animal model studies. Additionally, we identified only one human study evaluating apremilast (a phosphodiesterase 4 inhibitor) in patients with psoriasis. The results suggest that it may counteract bone resorption by modulating RANKL levels and osteoclast differentiation; however, no specific data on its effects on BMD are available (83).

## 6 Limitations

The studies analyzed have several limitations. Many are retrospective or non-randomized, and sample sizes—especially in the case of non-TNFi treatments—are small. Baseline characteristics are not always homogeneous, and most studies lack a comparator group. In addition, for some drugs, data on their effects on bone mass are scarce or absent. Most studies have focused exclusively on patients with RA, even though many of these treatments are also approved for other IMIDs. In turn, the follow-up duration in several studies may be insufficient to detect long-term effects on bone metabolism.

Furthermore, most of the available evidence is based primarily on BMD, while bone quality has been insufficiently assessed in IMIDs. Studies in RA and axSpA have shown deterioration of trabecular and cortical microarchitecture, even in the presence of preserved BMD values (84–86), highlighting the need to incorporate complementary measures such as advanced imaging techniques or the trabecular bone score (TBS). This tool, which can be used in clinical practice, predicts fractures. Some descriptive observational studies suggest that biologic therapies may have a beneficial effect on trabecular bone, as assessed by TBS, in patients with RA (87, 88).

Studies of factors associated with fracture risk did not include validated tools such as FRAX. FRAX is widely used in clinical practice to estimate the 10-year probability of major osteoporotic fractures and hip fractures. However, it has been noted that this tool may underestimate the risk of fracture in chronic inflammatory diseases, as it does not incorporate disease-specific factors such as inflammatory activity, structural damage, cumulative glucocorticoid load or the impact of other treatments (89, 90), which may limit its accuracy in patients with IMIDs.

Finally, confounding by indication is an important limitation of the available evidence. Patients receiving bDMARDs or tsDMARDs typically have more severe and longer-standing disease, higher cumulative exposure to glucocorticoids, and a greater inflammatory burden compared with those treated with conventional therapies. These factors independently influence BMD and fracture risk and may therefore confound the observed associations between treatment and skeletal outcomes. Given that most studies are observational, residual confounding cannot be excluded.

Taken together, these limitations highlight the need for well-designed, controlled studies with adequate statistical power, which incorporate the considerations outlined above, in order to fully elucidate the impact of bDMARDs and tsDMARDs on bone health in IMIDs.

## 7 Key clinical implications and practical recommendations

bDMARDs and tsDMARDs are commonly prescribed for patients with persistent inflammatory disease or disease refractory to conventional treatment, who are typically characterized by a high inflammatory burden and greater cumulative exposure to glucocorticoids. Both factors independently contribute to BMD loss and an increased risk of fracture.

BMD assessment by dual-energy X-ray absorptiometry should be interpreted with particular caution in patients with axSpA. In these patients, the presence of syndesmophytes and spinal ossification may lead to overestimation of LS BMD, thereby limiting its clinical utility. Consequently, hip BMD should be preferentially considered, and vertebral fractures should be assessed systematically. In addition, a more comprehensive evaluation of bone health may include assessment of bone quality using TBS, particularly in patients with risk factors for skeletal fragility in whom BMD does not adequately reflect fracture risk.

The currently available evidence does not support the routine measurement of BTMs in clinical practice for the assessment or monitoring of bone health in patients with inflammatory diseases receiving bDMARDs or tsDMARDs. Their use may be restricted to specific situations, such as monitoring the response to anti-osteoporotic therapy or in selected research settings; however, the available evidence is insufficient to support their routine use in this population.

The available evidence suggests that preservation of bone health should be regarded as an additional benefit of bDMARDs and tsDMARDs, primarily resulting from effective control of inflammatory disease activity and reduced cumulative glucocorticoid exposure, rather than as a primary therapeutic objective. Although several agents preserve or improve BMD, current evidence does not justify modifying osteoporosis assessment strategies or fracture prevention approaches according to the specific bDMARD or tsDMARD used.

Bone health should be assessed in accordance with established recommendations for osteoporosis and the individual’s overall fracture risk, particularly in patients with persistent inflammatory disease activity, prolonged glucocorticoid exposure, a history of fragility fractures, older age, or other conventional risk factors for osteoporosis. Anti-osteoporotic treatment should be initiated whenever indicated, regardless of the bDMARD or tsDMARD regimen being used.

## 8 Conclusion

Our review showed that, in general, bone mass is maintained or increased with the use of bDMARDs and tsDMARDs. However, decreased BMD has been reported, usually in association with persistent inflammatory disease activity. The behavior of BTMs is variable and does not always correlate with changes in BMD. Importantly, improvements in BMD do not necessarily translate into a reduced fracture risk, and, to date, none of these treatments have consistently demonstrated a reduction in the incidence of fracture. Only studies conducted in patients with RA suggest that TNFi could maintain fracture risk at a level similar to that of patients with less active disease who do not require biologic therapy. This may be explained by the fact that BMD primarily reflects bone mass quantity and does not fully capture bone structural quality. Furthermore, factors such as accumulated bone damage, comorbidities, and other unmodified risk factors significantly influence the probability of fracture.

These findings highlight the importance of effective inflammation control for bone preservation and the need to evaluate both bone quantity and bone quality when assessing skeletal health. From a clinical perspective, the choice of bDMARD or tsDMARD should not modify the standard approach to osteoporosis assessment or fracture prevention. Bone health should continue to be evaluated according to individual fracture risk, particularly in patients with persistent disease activity, glucocorticoid exposure, previous fragility fractures, or other established risk factors for osteoporosis. In patients with axial spondyloarthritis, lumbar spine BMD should be interpreted with caution because structural spinal changes may overestimate bone mass. Evidence is still limited for non-RA IMIDs, and long-term, prospective, controlled studies are required to better define the impact of bDMARDs and tsDMARDs on bone health and fracture risk across different patient populations.

## 9 Concluding remarks and future perspectives

### 9.1 Limited evidence beyond rheumatoid arthritis

Most studies focus on RA, even though many bDMARDs and tsDMARDs are approved for other IMIDs such as PsA, axSpA, and IBD. Future studies should evaluate bone outcomes in these populations.

### 9.2 Need for long-term controlled trials

Evidence is largely from retrospective or small prospective studies, often without comparator groups. Well-designed, long-term, randomized trials are needed to assess the sustained effects of these therapies on bone mass and fracture risk, systematically incorporating all factors that may influence bone health—including inflammatory activity, structural damage, cumulative exposure to glucocorticoids, comorbidities and other determinants of fracture risk—in order to ensure an accurate interpretation of the results.

### 9.3 Discrepancy between bone density and fracture risk

While some treatments improve BMD, their effect on fracture risk remains unclear. Future research should incorporate clinical fracture endpoints, not only densitometry, and explore bone quality markers, including microarchitecture, bone strength, and assessments such as the trabecular bone score, to provide a more comprehensive evaluation of skeletal health.
